# Response of Fibroblastic Rheumatism to Infliximab

**DOI:** 10.1155/2009/715729

**Published:** 2010-02-01

**Authors:** Ricardo Romiti, Mauricio Levy Neto, Marcello Menta Simonsen Nico

**Affiliations:** Departments of Dermatology and Rheumatology, University of São Paulo, 05403-000 São Paulo, SP, Brazil

## Abstract

Onset is usually sudden with symmetrical articular involvement of both small and large joints, occurrence of solid skin nodules, and rapid progression. Treatment is generally unrewarding. Here we report a severe and disabling case of FR responding favorably to infliximab therapy. After 32 weeks of continuous treatment, skin lesions dramatically improved and arthropathy partially regressed.

Fibroblastic rheumatism (FR) is a rare disease of unknown cause, initially described by Chaouat et al. in 1980. 

The clinical picture of FR consists of skin nodules and polyarthropathy with flexion contractures of the fingers. Histological and immunohistochemical findings suggest that FR should be included in the group of fibromatoses. Our group recently reported a case of FR with exuberant clinical findings [1]. The patient was a 40-year-old man that presented with a 3-month history of cutaneous nodules. After a few weeks, his skin acquired a severe sclerodermoid aspect and significant impairment resulted. Magnetic resonance of the hands showed tenosynovitis of the flexors, synovitis of the metacarpophalangeal joints, and osteoarthritis of the thumbs. Diagnosis of FR was based established on clinical and pathological findings.

The patient was treated with prednisone (0.4 mg/kg/day) with mild cutaneous improvement. Due to progressive worsening of his articular deformities, the dose of prednisone was increased to 1.0 mg/kg/day and methotrexate, 25 mg/week, was added. After 6 months, as no response was noted, the TNF-alpha inhibitor, infliximab, was started. Therapeutic regimen consisted of infliximab, 3.0 mg/kg, in combination with methotrexate—the same regimen used for treating rheumatoid arthritis. After the first three doses of infliximab at weeks 0, 2, and, 6, significant reduction of nodules could be observed. After 22 weeks of maintenance treatment, skin fibrosis decreased and was accompanied by moderate improvement of joint mobility (Figures 1(a) and 1(b)). Additionally, the patient could now perform several manual activities that were impossible before. After an additional 10-week treatment period, clinical response persisted and no side-effects were reported.

FR is acknowledged as very difficult to treat. Therapeutic modalities have included prednisone, colchicine, interferon, penicillamine, methotrexate, and nonsteroidal anti-inflammatory drugs, with mostly poor results.

TNF-alpha is a proinflammatory cytokine released by macrophages, monocytes, and T lymphocytes as well as many tissue-specific cell types including keratinocytes and dendritic cells. The clinical effectiveness of the TNF-alpha inhibitor, infliximab, in the treatment of different inflammatory disorders has been demonstrated in several studies. Although psoriasis remains the only condition in dermatology for which the use of biologic immunomodulators has been approved, different disorders have been reported to favorably respond to anti-TNF treatment, including hidradenitis suppurative, pyoderma gangrenosum, as well as the noninfectious granulomatous diseases, among others [2]. It is known that TNF alpha participates in activation of vascular endothelium, regulation of immune response, and metabolism of the connective tissue by modulation of fibroblastic function. Blockage of TNF alpha could possibly explain partial regression of sclerodermoid alterations in FR treated with infliximab. Our experience suggests that the spectrum of disorders responding favorably to anti-TNF agents may further include disabling FR. Other reports are necessary to definitely confirm the efficacy of this novel therapeutic indication of infliximab.

## Figures and Tables

**Figure 1:**
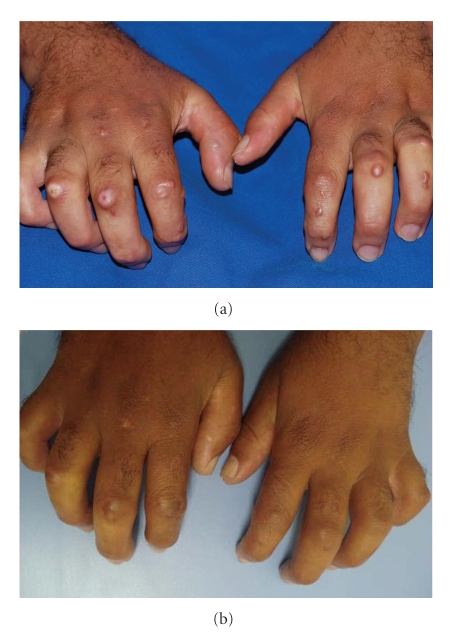
(a) Fibroblastic rheumatism before treatment. (b) Improvement after 22 weeks, showing visible reduction of all lesions.
